# Pregnancy Loss (28–110 Days of Pregnancy) in Holstein Cows: A Retrospective Study

**DOI:** 10.3390/ani10060925

**Published:** 2020-05-26

**Authors:** Aitor Fernandez-Novo, Octavi Fargas, Juan Manuel Loste, Francisco Sebastian, Natividad Perez-Villalobos, Jose Luis Pesantez-Pacheco, Raquel Patron-Collantes, Susana Astiz

**Affiliations:** 1 Dpto. Producción y Sanidad Animal, Salud Pública Veterinaria y Ciencia y Tecnología de los Alimentos, Facultad de Veterinaria, Universidad CEU Cardenal Herrera, C/Tirant lo Blanc, 7, 46115 Alfara del Patriarca, Valencia, Spain; aitor.fernandeznovo@uchceu.es; 2 VAPL S.L., C/Antoni Figueras 20, 08551 Tona, Barcelona, Spain; octavi.vaplslp@gmail.com; 3 Akaborro s/n., 31860 Navarra, Spain; loste@albaitaritza.com; 4 Cowvet SL, Avda. País Valenciano 6, 5, 46117 Betera, Valencia, Spain; cowvetsl@gmail.com; 5 Facultad de Ciencias Biomédicas, Universidad Europea de Madrid, C/Tajo s/n, 28670 Villaviciosa de Odón, Madrid, Spain; natividad.perez@universidadeuropea.es; 6 School of Veterinary Medicine and Zootechnics, Faculty of Agricultural Sciences, University of Cuenca, Avda. Doce de Octubre, 010150 Cuenca, Ecuador; jose.pesantez@ucuenca.edu.ec; 7 TRIALVET S.L., C/Encina 22, Cabanillas de la Sierra, 28721 Madrid, Spain; rpatron@trialvet.com; 8 Dpto. Reproducción Animal (INIA), Avda. Puerta de Hierro s/n., 28040 Madrid, Spain

**Keywords:** reproductive strategy, parity, season, rank of AI, type of AI

## Abstract

**Simple Summary:**

High-yield dairy cow farms have implemented high technified management for the last few decades, aiming at optimizing productions with the best animal welfare canons. A key point to achieve this is the reproductive performance. Around 12% of cattle suffer pregnancy loss during the late embryonic/early foetal period (between 28 and 110 day of pregnancy). Thus, our objective was to study the pregnancy losses occurring in eight different Spanish high-yielding Holstein dairy herds, in locations with severe heat stress during the summer, to examine the link between pregnancy loss and different management factors. Some factors, previously confirmed as significant ones, such as the technician who performed artificial insemination (AI), fixed-time or after observed oestrus AI, the bull used, type of semen or season, did not affect pregnancy loss in our study. Moreover, older cows (compared to heifers), first artificial inseminations (compared to ≥2nd ones) and pregnancies after fixed-time-AI (compared to AI after observed oestrus and natural breedings) were definitively associated to higher pregnancy loss. Therefore, farmers and consultants should adapt their prevention strategies relating to pregnancy loss, particularly, to the parity of the cattle and to type and rank of AI.

**Abstract:**

The objective of this retrospective study was to investigate the prevalence of pregnancy loss (PL; between 28–110 pregnancy days) and its relationship with factors: farm, year (2015–2018), season, artificial insemination (AI)-rank, parity, AI-type (fixed-time vs. oestrus-AI), previous PL, days in milk (DIM), fixed-time-AI protocol, AI-technician, bull, and semen-type (sexed vs. conventional). Data of 19,437 Holstein cattle AIs from eight Spanish farms were studied. Overall conception rate was 34.3% (6696/19,437) and PL 12.3% (822/6696). The PL was more likely to occur in primiparous (10.8%, odds ratio (OR) = 1.35; *p* = 0.04) and multiparous (15.3%; OR = 2.02, *p* < 0.01) than in heifers (PL = 6.9%, reference). Pregnancies achieved with AI after observed oestrus and natural breedings were associated with less PL than pregnancies after fixed-time-AI (12.7 vs. 11.9%; OR = 0.12, *p* = 0.01). First AIs related to higher PL than ≥2nd AIs (PL = 13.8% vs. 11.2; OR = 0.73, *p* < 0.01). The factors season, fixed-time-AI protocol, DIM, bull, AI-technician, or type of semen were not significantly associated with PL. Therefore, farmers and consultants should adapt their preventive strategies relating to PL, particularly, to the parity of the cattle.

## 1. Introduction

Pregnancy loss (PL) during the late embryonic and early fetal phases of pregnancy is a major cause of infertility in dairy cows and a crucial factor for dairy cattle farms’ economy [1,2]. Embryonic losses are categorized as early or late before and after day 25, respectively [1], the average rate of late embryonic loss being 10–12%, with this rate ranging broadly among farms from 3.5% to 26.3% [3].

Some studies have focused on possible predictors to forecast pregnancy loss [4], while other studies have concentrated on contributing factors. The following PL risk factors have been observed in epidemiological studies: parity, with multiparous showing higher PL than younger cows [5,6]; individual bulls [1,7,8,9]. Progesterone level at AI and during the first days of pregnancy has also been related to PL [10,11,12]. Similarly, the serum concentration of pregnancy-associated glycorproteins (PAGs) [13,14] is correlated to PL probability; the intensity of oestrus at AI [12,15], season at AI [16,17], the protocol for timed AI (FTAI) [18,19], twin pregnancy [9], and ovulation number [12]. Some other factors were not associated with pregnancy loss, such as the rank of AI [16], extended lactation [20], calving-AI interval [16], corpus luteum, and uterine blood flow at first pregnancy diagnosis observed with ultrasonography [10]. However, information is contradictory, and there are other studies that did not outline significant links between these factors and pregnancy loss [21,22].

In light of these observations, it is generally accepted that pregnancy loss in high-producing dairy cows is multifactorial, yet the exact underlying mechanisms remain unknown [3,23,24]. Previous reports have described the incidence and pattern of pregnancy loss in dairy cattle [25], but several of these studies assessed a limited number of cows or included the phases of pregnancy loss (from fertilization to pregnancy term) without clearly separating them [6,12,26]. Therefore, further information on this topic—clarifying factors and pregnancy phases, including a large number of pregnancies and exploring new risk issues—would be welcome, and even crucial, in helping us to find answers to practical questions, and guide management strategies towards improving reproductive efficiency in dairy farms. The hypothesis of the authors is that having lost a previous pregnancy is not a risk factor for future PL, while other factors such as farm, season, or AI rank play a key role. Therefore, the objective of this observational, retrospective study was to examine, in an extensive sample of pregnancies in high-yielding dairy cows, the relationship between pregnancy loss prevalence and the different factors described.

## 2. Materials and Methods

### 2.1. Ethics Statement

This is an observational, retrospective study based on data provided by collaborating veterinarian consultants relating to AIs and natural breedings performed at eight high-yielding, intensively managed dairy cattle farms located in different Spanish regions. No experimental intervention was performed, and farms carried out all their activities according to the EU Directive about the protection of animals in animal production [27].

### 2.2. Herds and Management

The study included a total of 19,437 AIs from dairy cattle and heifers (Holstein breed), performed during the period 2015–2018, from eight different intensively managed Spanish farms located in central and eastern Spain, where heat stress during the summer (June to August) is severe. Data taken from farm computers was provided by veterinary consultants. Data on heifers was provided only by Farms 4, 7, and 8.

Average milk yield and farm size were 34.8 ± 2.3 L/cow/day (range of 31.3 to 37.9 L/cow/day) and 746 ± 571 cows/herd (range of 253 to 1950 cows/herd). All herds were managed under similar conditions, being milked three times per day, with ad libitum access to water, and fed twice daily with a total mixed ration that was balanced to meet or exceed nutrient recommendations for lactating dairy cows. All farms are located in areas with severe heat stress during the summer, and cooling methods, such as fans with sprinkler systems on feeding and resting barns, were counted.

Reproductive management varied across farms, but in general, the farms used one long synchronization protocol for first AIs (Presynch-Ovsynch, Double Ovsynch, or G6G), and AI after observed oestrus for subsequent AIs if possible. If not detected in oestrus, all farms applied shorter synchronization protocols for 2nd or subsequent AIs (Ovsynch, 5d-Ovsynch, 5d-Cosynch, and 5d-Cosynch with intravaginal progesterone device). Pregnancy diagnoses were performed in all farms with ultrasound at 28–35 days for first pregnancy diagnosis, and between 100 and 110 days for second pregnancy diagnoses by the same experienced veterinarians at each farm. A positive diagnosis of pregnancy was made if the allantochorion and embryo in the uterine lumen were visualized and a heartbeat was detected, with ovaries being scanned only if problems arose when imaging the conceptus. On Farm 8, natural breeding was used for “repeating cows” with more than five AIs. In the case of heifers, farms implemented oestrus synchronization protocols with intravaginal progesterone device, maintained during 7 d with GnRH administration at the day of IPD insertion and prostaglandin at the day of IPD removal, or with prostaglandin injections 7 d apart, and AI after observed oestrus. Farm 8 included a natural service for repeating heifers after two failing AIs. Detailed information regarding farm, protocol, and AIs is summarized in Table 1 and Table 2.

### 2.3. Recorded Data

The following information was recorded for each AI or service: Farm (1 to 8); animal identification; year of AI; AI date (seasonality at service); rank of AI (1st to 11th); lactation order of the cow from 0 to 7th lactation and categorized as heifer, primiparous, or multiparous; reproductive strategy; days in milk; type of AI (fixed time AI with hormonally controlled follicle development vs. AI after observed oestrus and natural service, with natural follicle development (FTAI vs. OE + NS)), AI-technician, type of semen (conventional or sexed shorted seminal doses) and bull. Finally, in all AIs included, the factor if a pregnancy loss had occurred in the same lactation prior to the AI under consideration (previous PL in the same lactation, yes or not), and if the animal had suffered PL at least once before when considering all subsequent AIs, during the same and following lactations (previous PL during its lifetime, yes or no) was studied. Days in milk at service was logically only available in cows (40 to 848 d for first and further AIs); AI technician and type of semen (if sexed or conventional semen was used) was available only in farms 7 and 8; 9829 AIs); age at service in heifers was available only for heifers’ AIs from farm 4 (716 services; 399 to 668 d of age);

The number of AIs included in the study was 812/19,437 from Farm 1 (4.2%); 710/19,437 from Farm 2 (3.7%); 1865/19,437 from Farm 3 (9.6%); 4103/19,437 from Farm 4 (21.1%); 1530/19,437 from Farm 5 (7.9%); 588/19,437 from Farm 6 (3.0%); 3368/19,437 from Farm 7 (17.3%); and 6461/19,437 (33.2%) from Farm 8.

Synchronization protocols recorded for timed AI (FTAI) were as follows:

PRES or Presynch-Ovsynch protocol, as described by Moreira et al. [28]. In brief, cows received 2 injections of PGF2α administered 14 d apart beginning 26 d before initiation of Ovsynch-FTAI protocol, which consists of GnRH1 on day 0, PGF2α on Day 7, GnRH2 on day 9 and FTAI 16 h after last GnRH. The authors included the modification of two prostaglandins 24 h apart before the last gonadotropin-releasing hormone or GnRH2; (total of FTAIs included *n* = 544; 2.8%); DOV: Double Ovsynch described by Souza et al. [29]. Briefly, treatment with GnRH, followed 7 d later by PGF2α, then 3 d later a GnRH treatment, and finally by the Ovsynch-FTAI protocol. Authors included the modification of two prostaglandins 24 h apart before the last GnRH (total of FTAIs included *n* = 4534; 2.3%); G6G described by Bello et al. [30]. In short, this protocol included presynchronization with PGF2α, followed 2 d later with GnRH, and then 6 d later with the first GnRH injection of the Ovsynch-FTAI protocol. Again, authors included the modification of two prostaglandins 24 h apart before the last GnRH [31] (total of FTAIs included *n* = 3332; 17.1%); OVS: classical Ovsynch [32], described before, with the modification of two prostaglandins 24 h apart before the last GnRH [33]; (total of FTAIs included *n* = 2490; 12.8%); OVS-IPD: classical Ovsynch with insertion of an intravaginal progesterone device between days 0 and 7 of the synchronization [34]; (total of FTAIs included *n* = 455; 2.3%); 5dCO or 5 d Cosynch, following Santos et al. [35]. Briefly, d 0 GnRH, d 5 and 6 PGF2α, d 9 GnRH and FTAI; (total of FTAIs included *n* = 401; 2.1%); 5dCO-IPD or 5 d Cosynch, identical to the previous protocol, with insertion of an intravaginal progesterone device between d 0 and 5 of the synchronization [36]; (total of FTAIs included *n* = 560; 2.9%); 5dOVS: 5 d Ovysnch or short Ovsynch. In brief, d 0 GnRH, d 5 and 6 PGF2α, d 7 GnRH and TAI 16 h after the last GnRH [35,37] (total of FTAIs included *n* = 587; 3%).

Natural service (NS; total services included 245; 1.3%) and AIs after observed oestrus (OE; total AIs included 10,370; 53.4%) were included as further reproductive strategies. Therefore, ten reproductive strategies were included in the analysis, the eight firstly described for FTAI, the natural service, and AI after OE.

Average Days in Milk (DIM) at first FTAI in cows was 86.13 ± 39.33 d, and the average age of heifers at first service was 438 ± 27.9 d. Average DIM at any service was 149.9 ± 87.75 d, ranging among farms from 114 ± 51.4 in Farm 2 to a maximum of 166 ± 103.7 in Farm 8, and the average age of heifers at service (716, all from farm 4, the only one to provide this data) was 459 ± 44.7 d.

A total of 7094 services/AI were first AIs (36.5%), and 12,346 were 2nd or subsequent AIs, of which 3277 (16.9%) were 5th or subsequent AIs. A total of 8822/19,437 were FTAI, and 10,615/19,437 were AI after observed oestrus and natural services. The AIs were performed in animals with different lactation orders, with 2557 services/AIs having been in heifers (13.1%), 5965 in primiparous cows (30.7%), and the rest (10,936 AIs; 56.2%) in multiparous cows (with lactation order up to 7th). From the total of 2557 services performed on heifers, 867 (34%) came from Farm 4, 716 (28%) from Farm 7, and 974 (38%) from Farm 8. All farms provided data on services on primiparous and multiparous animals, including data on lactation order 0 (heifers) to the 7th lactation.

A total of 14,629 breedings/AIs (75.3%) were performed during the cool season (September to May) and 4808 (24.7%) during the hot season (June, July, and August). The breakdown during the period of study was 2282/19,437 (11.7%) in 2015; 10,442/19,437 (53.7%) in 2016; 5443/19,437 (28%) in 2017; 1270/19,437 (6.5%) during 2018.

Frozen semen from 216 different bulls was used in the different farms, plus the four bulls used for natural mounting (Farm 8). From the total of 9829 AIs with information regarding if sexed or conventional semen was used, 1026 AIs were performed using sexed, frozen, commercial semen. A total of 16 experienced AI technicians/veterinarians performed all AIs during the time of data recording. Table 1 and Table 2 show the breakdown of the AIs and services provided by farm, reproductive strategy followed, and animal type (heifer, primiparous, or multiparous).

Wiltbank et al. [3] described four pivotal periods of pregnancy when studying pregnancy loss from fertilization failure to fetal loss. In brief, the first period occurs during the first week after breeding with the absence of fertilization or death of the early embryo, with 20–50% of dairy cows experiencing pregnancy loss during this period. Key factors here are oocyte quality and progesterone concentrations during the preovulatory phase (with issues such as heat stress, inflammatory diseases, body condition being essential). The second pivotal period (days 8 to 27), covers embryo elongation and the maternal recognition of pregnancy, with losses around 30%, with a huge variation between farms. Maintenance of the corpus luteum (CL) of pregnancy is the key factor here. The third pivotal period (days 28 to 60), with losses of 12%, seems to depend on delays or defects in placentomes or embryo. The fourth period covers the third month of pregnancy and show a reduced pregnancy loss incidence (2%), that can be high in cows carrying twins in the same uterine horn. The current study aims to include pregnancy losses occurring in pivotal periods 3 and 4, corresponding to the phases of “late embryonic and early fetal losses” described in previous studies [5]. Consequently, we included all PL cases documented after a positive pregnancy diagnosis (>28 and <35 d of pregnancy) and with a further negative pregnancy diagnosis at days >70 and <110 after AI/service.

In order to determine whether a cow which had previously lost its pregnancy showed a different probability of getting pregnant and losing pregnancy during the rest of its life in services following that first PL, animals were classified according to whether they had lost a pregnancy at least once in their life, compared with those cows with no PL history (including only animals whose full history was known, and including the whole reproductive history of all these cows; *n* = 5614 services). An additional analysis sought to investigate whether an animal that had experienced PL changed its future probability of getting pregnant and losing pregnancy after subsequent AI/services during the same lactation after the previous PL.

### 2.4. Conception Rate: Descriptive Results

Conception rate (CR) was not the aim of the present study. Therefore, only descriptive results are outlined. The overall conception rate observed was 34.3% (6696/19,437). Descriptive data on CR values classified by reproductive strategy, farm, and other factors is summarized in Table 3.

### 2.5. Statistical Analyses

All data were analyzed using SPSS^®^ 25 (IBM, Armonk, NY, USA). Probability values less than or equal to 0.05 were considered significant, and those between 0.05 and 0.1 were considered trends. All data are reported as a mean percentage (categorical variables) or as mean ± SD (numeric variables). Pregnancy loss probability was analyzed using logistic regression, odds ratios (OR) were determined, and a stepwise forward method based on the Wald statistics criterion of *p* > 0.10. The statistical model included farm, animal, and year as random effects to control the effect for repeated AIs.

Different subsets of data were analyzed separately in different regression models in order to control the reduction of the size of the total population analyzed. A first, main, analysis was performed including all pregnancies analyzed (*n* = 6696 and a total of pregnancies lost 822) and the following factors (available in all pregnancies): farm, animal, year, season, AI-rank, lactation order (categorized in heifers, primiparous, or multiparous), type of AI (fixed-time AI with hormonally controlled follicular development vs. AI after observed oestrus and natural service, with natural development of the follicle (FTAI vs. OE + NS)), reproductive strategy, and previous pregnancy loss. Information on type of semen used (if sexed or conventional semen) and AI-technician was only available in data coming from Farms 7 and 8. Therefore, an additional analysis was performed on these 3141 pregnancies, including all factors previously detailed. A third regression model was conducted on the subset of pregnancies coming from cows (excluding heifers) with a total of 5408 pregnancies, including the additional factor of average Days in Milk (DIM) at each AI. A fourth analysis was performed, including only heifers (*n* = 1288 pregnancies) with the factors: farm, animal, year, season, AI-rank, type of AI, reproductive strategy, and previous pregnancy loss. The last regression model conducted was on a reduced subset of data of heifers’ pregnancies, including the additional factor and age at AI, only provided by Farm 4 (716 AIs). In all the models the following interactions were explored: reproductive strategy × farm; reproductive strategy × season; reproductive strategy × parity; reproductive strategy × type of semen, reproductive strategy × rank of AI, and type of AI × farm. Once demonstrated that interactions were not significant, the second phase in each analysis was performed without those not significant interactions.

## 3. Results

From a total of 6696 diagnosed pregnancies after a first pregnancy diagnosis and <day 110 of pregnancy (between 28–110 days after AI), a general pregnancy loss rate (PL) of 12.3% was found (822/6696). A PL percentage of 11.9% was observed during the cool season (636/5360) and 13.9% during the hot season (186/1336; *p* = 0.32). Regarding the order of AIs or AI-rank, the PL detected was 13.8% for pregnancies induced by first AIs (372/2692; *p* < 0.01), 11.1% for second services (353/3184), and 11.8% for third and subsequent services (97/820). Heifers achieved the lowest PL (6.9%; 89/1288), followed by primiparous cows (10.8%; 223/2066; *p* = 0.04), while multiparous cows showed the highest PL rate (15.3%; 510/3342; *p* < 0.01; difference primiparous vs. multiparous with *p* > 0.05).

PL results distributed for each type of reproductive strategy across the eight farms are summarized in Table 4. When segmenting the data and observing only heifers, the pregnancy loss rate by protocol was 0.5% (1/194) for natural service, 7.7% (76/982) for observed oestrus, 33.3% (2/6) for OVS, and 9.6% (10/104) for OVS-IPD.

### Logistic Regression Models on Pregnancy Loss Probability, Including all Recorded Factors and Different Data Subsets

The logistic regression analysis results on pregnancy loss, including all pregnancies studied (n = 6696), are summarized in Table 5. As described previously, individual animal, year and farm were introduced in the model as random effects. The analyses determined that the *P*-values were *p* = 0.47 for farm; for the factor animal: *p* = 0.06 and for the interactions reproductive strategy × parity, reproductive strategy × AI rank and AI type × farm, *p* = 0.58, *p* = 0.87 and *p* = 0.30, respectively. Therefore, only the factor year was kept in the final model (Table 5).

Second or more AIs, being a heifer and AI after observed oestrus or natural breeding, were associated with a lower PL rate, while not having a PL history during the same lactation tended to be associated with higher PL. The factors season, previously having lost a pregnancy in life and reproductive strategy, were not associated with PL (*p* > 0.05).

Having had a previous PL in the lactation, tended to have a reduced probability of losing pregnancy again during the same lactation (780/6253, 12.5% vs. 42/443, 9.5% of PL for cows without and with PL history, respectively; OR = 0.77; 95% CI 0.551–1.086; *p* = 0.13).

No relationship to PL was detected for the different hormonal protocols for fixed time AI and AI after observed oestrus (*p* > 0.05).

The sub-model observing only cows (total pregnancies: 5408), in order to analyse the same model adding days in milk, demonstrated that DIM did not influence PL (*p* = 0.76). Subsequently, factors not provided by all farms were added, such as AI-technitian and type of semen used. The model observing AI-technician and type of semen, provided only by Farms 7 and 8, included 9829 AIs and 3141 pregnancies (408 pregnancies after using sexed semen and 2733 after using conventional semen). In this model, AI-technician was not associated with pregnancy loss (*p* = 0.90), and neither was type of semen (*p* = 0.62). Pregnancy loss after the 3141 pregnancies wth sexed semen was 8.1% (33/408), while PL was 14.3% (391/2733) in pregnancies achieved with conventional semen. In this data subset, similarly to the subset of all included AIs, second or subsequent AIs were associated with lower PL compared with first services (*p* = 0.04; OR = 0.79; 95% CI: 0.63–0.99). Being a heifer as opposed to an adult cow was associated also with lower PL (OR = 2.16; 95% CI: 1.54–3.03 for primiparous and OR = 3.11; 95% CI: 2.27–4.26 for multiparous; *p* < 0.01). Having lost pregnancy related to a lower PL value in future pregnancies during the same lactation (OR = 0.62; 95% CI: 0.40–0.98; *p* = 0.04). No explored interaction and neither type of AI or season were significant factors for PL in this subset of AIs in cows.

Finally, the logistic regression model on the subset of heifers (total of AIs included 2557 and 1288 pregnancies) detected the factors Natural Service (PL = 0.5%; 1/194) and Ovsynch reproductive strategy (PL = 40%; 2/5), as associated with PL (OR = 0.004, 95% CI: 0.00–0.27, *p* = 0.010, and OR = 8.32, 95% CI: 1.30–53.23, *p* = 0.03, respectively).

## 4. Discussion

This study indicates a 12.3% overall rate of pregnancy loss between days 28 and 110 of pregnancy across all AIs and services included. The global PL rate observed (12.3%) in the present study is consistent with the 11.95% based on analysis of 24,391 pregnancies by Wiltbank et al. [3]. The hypothesis of the authors was that having lost previously pregnancy was not a risk factor for future PL. However, farm did note play a key role, while AI-rank and AI-type were significantly associated with PL.

Although in the present study the “farm” factor itself was not significantly associated with different PL results, we could consider Farm 4 to be an intrinsic factor worsening overall reproductive efficiency. In fact, Farm 4 was the farm with the second worst average conception rate (31.4%; Table 3), and it showed the worse PL rates after three different strategies (observed oestrus, G6G, and Ovsynch; Table 4), which would support a negative influence of the farm itself on its overall reproductive efficiency. The “farm” factor includes different features together, such as health status, management practices, and environmental conditions, which worsens the average farm level of PL as stated by other authors [3,8]. The factor animal was included as random factor, to control the effect of repeated AIs in one same animal. However, it was not significantly associated and could be removed from the final model.

On the contrary, the factor year of study, was strongly associated with PL with a decreasing PL rate being observed with time. We could, therefore, argue a general improvement of the reproductive management in general, which seems to be associated also to this reduction of PL with time.

The hot season at AI is a known risk factor for lower fertility [9,16,17]. However, heat stress is reported to mainly influence failure in very early pregnancy phases, such as fertilization and “Pregnancy Pivotal Periods” n 1 and 2 after Wiltbank et al.’s [3] classification (days of pregnancy from fertilization to day 28). Accordingly, in our study, season was not significantly associated with pregnancy loss during Pivotal Periods 3 and 4. García-Ispierto et al. [5] demonstrated a strong association between PL and heat stress, with the likelihood of pregnancy loss being associated with a PL increase by a factor of 1.05 for each additional unit of the mean maximum temperature–humidity index from days 21 to 30 of gestation. This factor may be a complex one, and is probably directly related to an additional factor connected with PL, the unilateral twinning pregnancy [37], which is the main risk factor for PL during the fourth pivotal period of pregnancy (60 to 90 days of pregnancy) [3] and which is linked to season [38]. Unfortunately, there was no information available on twin pregnancies or laterality of double CL in our dataset. The interaction reproductive strategy x season did neither result significant in this study. In farms with a severe heat stress (as it was the case of the farms in the study), farmers avoid investing resources in inseminating at fixed time during the hot season. Due to this reason, the number of pregancies achieved during the summer with hormonal protocols was limited.

Being a heifer as opposed to cows related to lower PL values. These results partly agree with other earlier observations, which showed that parity is positively associated with PL (more PL in high parity cows) [5,6]. Our study shows an eight-point PL difference between multiparous cows and heifers (15.3 vs. 6.9% of PL; *p* < 0.01), which is similar to the results of Starbuck et al. [39] who reported that older cows maintained 9% fewer pregnancies than younger ones. While embryo survival rates are assumed to be similar in heifers and low- to moderate-producing dairy cows, this influence could be related to a parity-related effect and could also be a consequence of milk yield level [25]. Accordingly, an increase in milk yield during the first 60 days in milk can increase early embryonic loss by almost 5 points [12]. Unfortunately, production data were not available in our data set.

In this study the type of AI was associated with PL. Pregnancies produced after natural follicle development (OE + NS) were associated to less PL (12.7% vs. 11.9%). In other studies, the occurrence of oestrus at AI, using tail chalk and tail head patch, was associated with a reduction in pregnancy loss in dairy cows [40,41]. Furthermore, Pereira et al. [41] reported that the reduction in pregnancy loss in cows that expressed oestrus at AI occurred regardless of the diameter of the pre-ovulatory follicle and the hormonal protocol. Madureira et al. [15] elucidated that the actual factor related to increased CR and decreased PL could be the intensity of oestrus expression, irrespective of the reproductive strategy implemented. In the present work, the type of AI with less PL pregnancies included those pregnancies after natural breeding, which may be the deffinitive factor, supporting the results of Madureira et al. [15] because animals inducing bulls to mount and tolerating natural breeding express estrous adequately. Moreover, the factor reproductive strategy which included all hormonal protocols and AI after observed oestrus, was not significantly associated with PL in the current study. The strategy for detecting oestrus in our farms, was to use activity devices where a strong oestrus expression is not required for insemination, which could explain this result. It seems that progesterone is the final modulator linking the better expression of oestrus with a better fertility and reduced pregnancy loss afterwards. Progesteron leads oestrous expression of higher intensity and greater fertility, and, consistently, increased progesterone concentration post-AI sustains embryo and fetal development [42], probably due to changes in the progesterone receptor profile within the endometrium [43].

The result that ≥2nd AIs were associated with less PL could reflect the less stressing situation with the cattle having overcome the lactation peak (more days in milk). However, this may also reflect just the low probability of occurring PL, together with the reduced number of AIs of higher rank, which numerically results in a overall reduced prevalence of PL in further AIs. The same fact may be behind the result obtained in the current study that animals having had a previous PL in the lactation tended to have a reduced probability of losing pregnancy again during the same lactation (12.5% vs. 9.5% of PL for cows without and with PL history, respectively). Necessarily, when a cow has experienced PL previously, further AIs in the same lactation are of a higher rank. Certain pathological events, although less likely to occur, might suggest the involvement of a possible infectious etiology in some cases of pregnancy loss [44]. However, this hypothesis is not supported by the fact that this association with PL of having had previous PL was not significant when observing AIs during the rest of the animal’s life. The retrospective design of this study does not allow detection of protective factors, but based on these results, we can state that further AIs after a pregnancy loss event and higher rank of AIs are not associated with an enhanced probability of PL.

No relationship to PL was detected for the different hormonal protocols for fixed time AI and AI after observed oestrus. Similarly, the meta-analysis performed by Borchardt et al. [21] showed no PL difference before day 60 of pregnancy in cows inseminated after Presynch AI. Similarly, several authors have failed to link determined protocols to PL rates [21,45,46,47,48], although they specifically pointed out the limited number of herds with similar management practices and of pregnancies included in some of the previous studies, and, consequently, on the limited number of pregnancy losses analyzed. These circumstances, could be similar in the current work. Moreover, although the reproductive strategy for AI is directly linked to the AI-rank, with long protocols (PRES, DOV, and G6G) mostly used for first AIs [49], no significance was detected for the “AI rank × reproductive strategy” interaction on PL.

Regarding the relationship protocol and PL, observing only heifers, as opposed to all cows, gave rise to different results, with the Ovsynch protocol being associated with higher PL (*p* = 0.03). Heifers show higher individual fertility than cows [5], and it is well known that the Ovsynch protocol does not correctly synchronize the ovulation in dairy heifers [32], this also probably being the reason for a higher PL subsequently when pregnant, due to less functional CLs [23], as observed in lactating dairy cows [40]. Other factors included in the study, such as AI-technician, bull semen, sexed vs. conventional semen, or DIM, were not significantly associated with PL. However, based on the retrospective, epidemiological, observational design of the study, and the reduced sample size in these sub-subsets that included these factors, we cannot rule out the lack of statistical power to detect such effects.

## 5. Conclusions

Our results demonstrate that being a heifer, second or more breedings and AI after observed oestrus and natural breeding have a notable impact, being associated to less PL. Having lost pregnancy previously, was detected as a slighter influencing factor (statistical tendency), relating to less PI in future pregnancies in the same lactation. However, factors previously demonstrated as significant, such as season, bull, AI technician or type of semen, might not be associated with PL. Therefore, farmers can decide inseminating again cows which suffered previous PL and farmers and consultants should adapt their preventive strategies against PL to cattle parity. These results highlight again, the complex reproductive characteristics of the pregnancy loss event and the relevance of further studies on this issue.

## Figures and Tables

**Table 1:** Descriptive statistics for the type of reproductive strategy used in 19,437 breedings across eight Spanish Holstein farms.

Reproductive Strategy	Farms																Breedings by Strategy	
	1		2		3		4		5		6		7		8			
	AIs	%	AIs	%	AIs	%	AIs	%	AIs	%	AIs	%	AIs	%	AIs	%	AIs	%
PRES													364	10.8	180	2.8	544	2.8
DOV	132	16.3					10	0.2	190	12.4			121	3.6			453	2.3
G6G	84	10.3	31	4.4	628	33.7	1046	25.5	367	24.0	423	71.8			753	11.7	3332	17.1
OVS	155	19.1	28	3.9			978	23.8					401	11.9	928	14.4	2490	12.8
OVS-IPD	92	11.3					10	0.2							353	5.5	455	2.3
5dOVS									540	35.3	47	8.0					587	3.0
5dCO					401	21.5											401	2.1
5dCO-IPD					560	30.0											560	2.9
OE	349	43.0	651	91.7	276	14.8	2059	50.2	433	28.3	118	20.1	2482	73.7	4002	61.9	10,370	53.4
NS															245	3.8	245	1.3
Breedings by farm	812	4.2	710	3.7	1865	9.6	4103	21.1	1530	7.9	588	3.0	3368	17.3	6461	33.2	19,437	

AIs: artificial inseminations; PRES: timed AI after Presynch; DOV: timed AI after Double Ovsynch; OVS: timed AI after Ovsynch; OVS-IPD: timed AI after Ovsynch with intravaginal progesterone device; 5dOVS: timed AI after 5 days Ovsynch; 5dCO: timed AI after 5 days Cosynch; 5dCO-IPD: timed AI after 5 days Cosynch with intravaginal progesterone device; OE: AI after observed oestrus; NS: natural service.

**Table 2:** Breeding descriptive statistics for the type of reproductive strategy used in heifer, primiparous, and multiparous Holstein cattle across eight Spanish farms.

Reproductive Strategy	Cattle Type									Breedings by Strategy	
	Heifer			Primiparous			Multiparous				
	AIs	(n/N) %	% *	AIs	(n/N) %	% *	AIs	(n/N) %	% *	AIs	%
PRES	3	(3/544) 0.5	0.1	160	(160/544) 29.4	2.7	382	(382/544) 70.1	3.5	544	2.8
DOV				296	(296/453) 65.3	5.0	157	(157/453) 34.7	1.4	453	2.3
G6G				985	(985/3332) 29.5	16.5	2,347	(2347/3332) 70.4	21.5	3332	17.1
OVS	17	(17/2490) 0.6	0.6	802	(802/2490) 32.2	13.5	1,667	(1667/2490) 66.9	15.3	2490	12.8
OVS-IPD	267	(267/455) 58.7	10.4	70	(70/455) 15.4	1.2	118	(118/455) 25.9	1.1	455	2.3
5dOVS				159	(159/587) 27.1	2.7	428	(428/587) 72.6	3.9	587	3.0
5dCO				189	(189/401) 47.1	3.1	212	(212/401) 52.9	2.0	401	2.1
5dCO-IPD				262	(262/560) 46.8	4.4	298	(298/560) 53.2	2.7	560	2.9
OE	2074	(2074/10,370) 20.0	81.1	3024	(3024/10,370) 29.2	50.7	5,272	(5272/10,370) 50.8	48.3	10,370	53.4
NS	198	(198/245) 80.8	7.8	13	(13/245) 5.3	0.2	34	(34/245) 13.9	0.3	245	1.3
Breedings by cattle type	2557	(2557/19,437) 13.1		5965	(5965/19,437) 30.7		10,915	(10,915/19,437) 56.2		19,437	

% *: indicates the overall percentage of breedings after each reproductive strategy used within each cattle type (heifers, primiparous, and multiparous). For abbreviations, see Table 1.

**Table 3:** Conception rate (CR) descriptive statistics for farm, reproductive strategy, season, AI-rank, cattle type, type of AI, and previous pregnancy loss across eight Spanish Holstein farms.

Farm *n*	Pregnancies/AI	CR (%)	Reproductive Strategy	Pregnancies/AI	CR (%)
1	226/812	27.8	PRES	152/544	27.9
2	294/710	41.4	DOV	167/453	36.9
3	853/1865	45.7	G6G	1353/3332	40.6
4	1290/4103	31.4	OVS	687/2490	27.5
5	616/1530	40.3	OVS-IPD	143/455	31.4
6	276/588	46.9	5dOVS	224/587	38.2
7	1229/3368	36.5	5dCO	168/401	41.9
8	1912/6461	29.6	5dCO-IPD	237/560	42.3
			OE	3364/10,370	32.4
			NS	201/245	82.0
Season	Pregnancies/AI	CR (%)	AI-rank	Pregnancies/AI	CR (%)
Cool	5360/14,629	36.6	First AI	2692/7094	37.9
Warm	1336/4808	27.8	Second AI	3184/9066	35.1
			≥3rd AI	820/3277	25.0
Cattle type	Pregnancies/AI	CR (%)	Type of AI	Pregnancies/AI	CR (%)
Heifers	1288/2557	50.4	FTAI	3131/8822	35.5
Primiparous	2066/5965	34.6	OE and NS	3565/10,615	33.6
Multiparous	3342/10,915	30.6			
Previous PL/lactation	Pregnancies/AI	CR (%)	Previous PL/life	Pregnancies/AI	CR (%)
No previous PL	6253/18,055	34.6	No previous PL	1675/3947	42.4
Previous PL	443/1382	32.1	Previous PL	517/1667	31.0

PL: Pregnancy loss. FTAI: fixed-time artificial insemination. OE: artificial insemination after observed oestrus; NS: natural service. For remaining abbreviations, see Table 1.

**Table 4:** Pregnancy loss descriptive statistics for the type of reproductive strategy across eight Spanish Holstein farms.

Reproductive Strategy	Farms																PL by Strategy	
	1		2		3		4		5		6		7		8			
	n/N	%	n/N	%	n/N	%	n/N	%	n/N	%	n/N	%	n/N	%	n/N	%	n/N	%
PRES													21/109	19.3	14/43	32.6	35/152	23.0
DOV	5/50	16.3					1/2	50.0	12/89	14.5			4/26	15.4			22/167	13.2
G6G	3/24	12.5	1/16	6.3	33/346	9.5	69/413	16.7	24/130	18.5	21/197	10.7			23/227	10.1	174/ 1353	12.9
OVS	3/38	7.9	1/7	14.3			27/298	9.1					4/26	15.4	40/239	16.7	93/687	9.4
OVS-IPD	1/13	7.7					10	0.2							10/130	7.7	11/143	7.7
5dOVS									21/213	9.9	0/11	0.0					21/224	9.4
5dCO					19/168	11.3											19/168	11.3
5dCO-IPD					24/237	10.1											24/237	10.1
OE	9/101	8.9	23/271	8.5	11/102	10.8	66/577	11.4	15/184	8.2	9/68	13.2	131/989	13.2	157/1072	14.6	421/ 3365	12.5
NS															2/201	1.0	2/201	1.0
PL by farm n/N %	21/ 226	9.3	25/ 294	8.5	87/ 853	10.2	163/ 1290	12.6	72/ 616	11.7	30/ 276	10.9	178/ 1229	14.5	246/ 1912	12.9	822/ 6696	12.3

n/N indicates the number of pregnancy loss events/number of pregnancies. For the remaining abbreviations, see Table 1.

**Table 5:** Logistic regression statistics for pregnancy loss across eight Spanish Holstein farms.

Factors	Class	n/N	PL (%)	Odds Ratio	95% Confidence Interval			*p*-Value
Year	2015, reference	110/583	18.9					
	2016	500/3318	15.1	0.73	0.569	-	0.889	0.01
	2017	176/2402	7.9	0.32	0.246	-	0.428	<0.01
	2018	36/393	9.2	0.43	0.285	-	0.651	<0.01
Season	Cool, reference	636/5360	11.9					
	Warm	186/1362	13.9	1.10	0.912	-	1.319	0.32
AI. rank	First AI, reference	372/2692	13.8					
	≥2nd AI	450/4004	11.2	0.73	0.605	-	0.876	<0.01
Parity	Heifer, reference	89/1288	6.9					
	Primiparous	223/2066	10.8	1.35	1.019	-	1.803	0.04
	Multiparous	510/3342	15.3	2.02	1.546	-	2.654	<0.01
AI type	FTAI, reference	399/3131	12.7			-		
	OE + NS	423/3565	11.9	0.12	0.026	-	0.532	0.01

n/N indicates number of pregnancy loss events/number of pregnancies; AI: artificial insemination; PL: pregnancy Loss; FTAI: fixed-time AI; OE: AI after observed oestrus; NS: natural breeding. Reference factors within each class are those placed in first instance.
